# Disability assessment as an outcome measure: a comparative study of Nigerian outpatients with schizophrenia and healthy control

**DOI:** 10.1186/s12991-015-0079-6

**Published:** 2015-11-23

**Authors:** Adesanmi Akinsulore, Boladale M. Mapayi, Olutayo O. Aloba, Ibidunni Oloniniyi, Femi O. Fatoye, Roger O. A. Makanjuola

**Affiliations:** Department of Mental Health, College of Health Sciences, Obafemi Awolowo University, Ile-Ife, Osun state Nigeria

**Keywords:** Schizophrenia, Disability, Outpatients, Outcome, Nigerian

## Abstract

**Background and objective:**

Schizophrenia is a chronic mental disorder that leads to disability in several aspects of the individual’s personal, social, and occupational functioning. This study assesses and compares the level of disability among Nigerian outpatients with schizophrenia and healthy controls (HC).

**Methods:**

A comparative cross-sectional study among 100 schizophrenia outpatients with an ICD-10 diagnosis and 100 HC was conducted over a 4-month period. They completed a questionnaire containing the Zung’s Self-Rating Depression Scale (SDS), the World Health Organization Disability Assessment Schedule-Second Version (WHODAS-II). Symptoms of schizophrenia were assessed with the Positive and Negative Syndrome Scale (PANSS). Student’s *t* tests and Chi-square were used to compare patient with schizophrenia and healthy control. Pearson correlation and multiple linear regression analyses were used to assess the relationships of socio-demographic and clinical variable with disability.

**Results:**

The patients with schizophrenia reported greater disability than the HC on most of the disability domains of WHODAS-II. They also reported significantly higher mean Zung’s SDS scores than the HC. Depressive symptoms, negative symptoms, and PANSS total were significantly related to all the WHODAS-II domains. The disability summary score was significantly predicted by depressive symptoms, negative and positive symptoms of schizophrenia, number of active symptoms (relapse) of schizophrenia, and marital status [*F* (5, 94) = 23.90, *p* < 0.001].

**Conclusion:**

Schizophrenia is a disabling disorder that affects different aspects of a patient’s life. Treatment strategies that target these different aspects may help in reducing disability.

## Background

Outcome measurement in psychiatry is a multidimensional construct that involves several independent domains such as clinical symptoms and disability assessment [1]. Disability is an important outcome measure that involves dynamic interactions between an individual’s health conditions and environment (social and attitudinal), quality of life, and the level of stigma experienced by the person [2–4]. Disability is any restriction or the deterioration of the expected functioning of an individual in a particular society by the family or social group or by the affected individual [5]. Therefore, social factors such as gender, unemployment, and family awareness of the nature of illness may influence the evaluation and level of disability in schizophrenia [6].

Disability in schizophrenia affects several aspects of the individual’s personal, social, and occupational functioning [7, 8]. The affected domains of patient’s daily life include self-care, self-management, vocational and leisure activities, and social relationships [2]. In schizophrenia, disability has been related to four domains of dysfunction which are the positive symptoms, negative symptoms, cognitive impairment, and affective symptoms [9, 10]. Positive symptoms are symptoms such as delusions and hallucinations that are “added on” to a patient’s experience during the course of illness. Also, negative symptoms such as a motivation refer to symptoms that indicate a reduction of normal functioning in the patient.

It is reported that positive symptoms of schizophrenia poorly predict future occupational functioning in schizophrenia [11, 12]. On the other hand, negative symptoms of schizophrenia have been significantly associated with disability in family and social functioning [13] and have stronger impact on real-world functioning than other symptoms [14]. Disability in schizophrenia has also been reported to be affected by socio-demographic characteristics (such as sex, marital, and employment status), depressive symptoms and cognitive deficit [2, 15, 16]. Reports from developed countries showed that schizophrenia is quite debilitative resulting in personal disabilities that eventually leads to economic loss [2, 15]. In addition, patients with schizophrenia experience greater severity of disability than the healthy controls (HC) [15, 16].

However, there is paucity of scientific data on disabilities among patients with schizophrenia in Nigeria. The majority of studies of disability in mentally ill Nigerians were conducted among elderly patients with disorders other than schizophrenia [17–19]. Gureje and Bamidele [20] assessed the social, occupational, and residential outcomes of Nigerian patients with schizophrenia and found that substantial proportion of them reported a moderate to severe degree of disability in the area of occupation and social contact. In a recent study, Adegbaju and colleagues [21] compared disability among adult Nigerian patients with bipolar affective disorder and schizophrenia and found that patients with schizophrenia reported greater disability than bipolar patients. However, they did not evaluate the association between disability and severity of schizophrenia symptoms. Therefore, we undertook this study to assess and compare self-reported disability using the World Health Organization Disability Assessment Schedule-Second Version (WHODAS-II) [22] in outpatients with schizophrenia and symptoms of schizophrenia. This study also evaluated the impact of disability on the different domains of patient’s life and provided baseline information on disability level among patients with schizophrenia in Nigeria. In this study, we aimed to assess and compare the level of disability among Nigerian outpatients with schizophrenia and healthy control group as well as identify predictors of disability in schizophrenia.

## Methods

### Participants

A comparative cross-sectional study conducted in the Mental Health Clinic of the Obafemi Awolowo University Teaching Hospitals Complex (OAUTHC) in Ile-Ife, Osun State, South Western Nigeria. The catchment area of OAUTHC includes villages and towns in Osun state and parts of Ekiti, Ondo and Edo states of Nigeria. Enrolment of patients was planned on a 4-month period (January–April 2012), and for each patient, we also enrolled a healthy control (HC) matched for age, gender, and educational level. The patients aged 18–64 years were diagnosed using the International Classification of Diseases tenth edition (ICD-10) [23], and the Mini International Neuropsychiatric Interview (MINI), English Version 5.0.0 [24], was also used to confirm the diagnosis. Also, patient must have been followed up for at least 1 year and their last hospital admission must be at least 6 months or above before the date of assessment. Patients with history of significant co-morbid organic disease or mental retardation and significant physical illness such as severe hypertension and diabetes mellitus were excluded from the study. The healthy controls were randomly selected from the residents of Ife Central Local Government Area of Ile-Ife which is divided into eleven wards with several streets. Five wards were randomly selected using simple balloting technique and twenty HC were chosen from each ward. From the list of streets within the selected wards, the first residence was chosen through a random sampling technique and every other third residence was selected subsequently. Only one HC who met the inclusion criteria was selected in each residence until the required number for each ward was achieved. Potential HC were excluded if they ever had a history of psychiatric illness or a family history of psychiatric illness in a first degree relative and significant physical illness. The study was approved by Ethics and Research Committee of OAUTHC. Written informed consent was taken from all the patients and HC after the objectives of the study had been explained to them. The patients and HC completed the socio-demographic questionnaire, the Zung’s SDS, and the WHODAS-II. Patients were also interviewed with MINI and PANSS.

### Instruments

Mini International Neuropsychiatric Interview: The MINI was designed as a brief structured interview for the major axis 1 psychiatric diagnoses in the DSM IV and ICD-10. It has a current (for present symptoms) and a lifetime version (for retrospective diagnosis). The lifetime diagnosis version was used in this study. The instrument has been used in Nigeria [25, 26].

Socio-demographic and clinical characteristics questionnaire: This questionnaire was developed by the authors for this study and was used to collect information from patients as well as their case files. Discrepancies were clarified by detailed questioning of the patients and relatives. The socio-demographic characteristics are age, sex, educational level, marital status, religion, and employment status. The clinical details of patients included age of onset and duration of the illness, number of active symptoms (relapse) of schizophrenia, number of hospitalization due to illness, and dose of current antipsychotic in chlorpromazine equivalents according to standard formulae [27, 28].

Positive and Negative Syndrome Scale (PANSS): The PANSS is a 30-item instrument that gives scores on positive and negative symptoms as well as general psychopathology [29]. For each item, ratings were made on a 1–7 scale of symptom severity. Positive symptoms were assessed with 7 items, while another seven addressed negative symptoms and 16 items assessed general psychopathology symptoms.

Zung’s Self-Rating Depression Scale (SDS): This was developed by Zung [30] to assess the severity of depressive symptoms. It is a 20-item self-administered questionnaire graded with a 4 point likert scale (Never, occasionally, sometimes, mostly) for each question. The sum of scores (raw scores) for each respondent was converted to a 100 point scale with a score of less than 50 points classified as normal, 50–59 points mild depression, 60–69 points moderate depression, and 70 points and above severe depression.

World Health Organization Disability Assessment Schedule-Second Version: Disability was assessed using the 36-item self-administered version of WHODAS-II [22]. It measures the difficulty the individual has had with performing particular daily activities over a period of 30 days. It consists of 36 likert-formatted questions, divided into six domains: understanding and communicating (D1; six items); getting around (D2; five items); self-care (D3; four items); getting along with others (D4; five items); life activities (D5; eight items); and participation in society (D6; eight items). The final score is calculated using a standardized SPSS algorithm. There are two syntax versions which may be administered according to a respondent’s occupational status: a 36-item version for those currently working and a 32-item version for those not working. The final scores derived from both versions range from 0–100 with higher scores indicating greater severity of disability.

### Statistical analysis

Statistical analyses were performed using the statistical package SPSS version 17.0 (SPSS Inc., Chicago, IL, USA). The Student *t* test and Chi-square statistics were used to study the differences between the two groups. Correlations between disability and psychiatric symptoms were studied using Pearson’s product moment correlation co-efficient. Multiple linear regression analysis with the stepwise method was used to explore the predictors (socio-demographic and clinical variables) of the disability summary score (DSS) of WHODAS-II. The level of statistical significance was considered to be *p* < 0.05.

## Results

Of the 108 consecutive outpatients that met the inclusion criteria, only 100 patients were enrolled for the study (three patients did not agree, two patients did not complete the scales, and three patients did not meet the lifetime diagnosis according to MINI). The mean age of the patients with schizophrenia and the normal subjects in this study were 39.53 years (SD = 9.295) and 39.47 years (SD = 9.419), respectively. Males comprised 52.0 % and females 48.0 % of the subjects in both groups. Most of the patients (78.0 %) and HC (70.0 %) were Christians. Two-thirds (67.0 %) of the patients and HC had up to secondary education. The HC were significantly better than the patients with regard to employment status and income per month. Also, significantly more of the HC were married (Table 1).

**Table 1:** Socio-demographic characteristics of patients with schizophrenia and healthy controls

Variables	Schizophrenia (*n* = 100)	Healthy controls (*n* = 100)	Test of statistical difference		
			*X* ^2^/*t* value	df	*p* value
Mean					
Age (years ± SD)	39.53 (9.295)	39.47 (9.419)	0.045	198	0.900
Sex					
Male	52	52	0.000	1	1.000
Female	48	48			
Educational level					
Primary	20	20	0.000	3	1.000
Secondary	47	47			
Non-university tertiary	17	17			
University tertiary	16	16			
Marital status					
Single	52	25	32.31	2	<0.001
Married	26	66			
Separated/divorced/	22	9			
Widowed					
Religion					
Christianity	78	70	1.663	1	0.259
Islam	22	30			
Employment status					
Employed	63	92	24.115	1	<0.001
Unemployed	37	8			
Income per month^a^					
<$33	20	0	54.207	3	<0.001
$33–$66.7	15	4			
$66.8–$333.3	22	74			
≥$333.3	6	14			

*SD* standard deviation

^a^Schizophrenia (*n* = 63), healthy controls (*n* = 92)

Table 2 shows the mean scores of the clinical characteristics of patients with schizophrenia and antipsychotic drug doses in chlorpromazine equivalents. The patients reported significantly higher disability scores than the HC in all the WHODAS-II domains except in the “getting around” domain. The patients also reported significantly higher mean Zung’s SDS scores than the HC (Table 3). In the patients with schizophrenia depressive symptoms, negative symptoms and PANSS total were significantly related to all the WHODAS-II domains. Also scores in all the WHODAS-II domains were significantly related to general psychopathology scores except in the work activities domain. Positive symptoms were significantly related to the WHODAS-II disability summary score, understanding and communicating domain, getting along domain, work activities domain, and participation in society domain. Depressive symptoms, negative symptoms, and general psychopathology showed the highest correlations in the WHODAS-II disability summary scores, while the positive symptoms and PANSS total scores showed the highest correlations with the WHODAS-II domain six which is concerned with participation in the society (Table 4).

**Table 2:** Clinical variables of patients with schizophrenia (*n * = 100)

Variables	Mean	Standard deviation
Age at onset of illness (years)	27.76	7.97
Duration of illness (years)	11.76	7.56
Number of active symptoms (relapse)	3.20	1.518
Number of hospitalization	1.05	1.226
Antipsychotic drug use (CPZE in mg)	374.03	366.56
PANSS total	40.62	7.12
PANSS positive	11.81	3.59
PANSS negative	8.74	2.44
PANSS general psychopathology	20.07	3.57
Zung’s SDS	43.50	12.21

*CPZE* chlorpromazine equivalent, *PANSS* Positive and Negative Syndrome Scale, *Zung’s SDS* Zung’s Self-Rating Depression Scale

**Table 3:** Comparison of patients with schizophrenia and healthy controls

Variable	Patients with Schizophrenia (*n* = 100)		Healthy controls (*n* = 100)		*t* value
	Mean	SD	Mean	SD	
WHODAS-II (DSS)					
Disability summary score	28.08	11.59	8.34	8.23	13.88**
Understanding and communicating (D1)	24.90	16.22	7.05	10.40	9.26**
Getting around (D2)	7.37	13.42	7.00	11.17	0.22
Self-care (D3)	3.20	8.97	0.90	3.21	2.41*
Getting along (D4)	44.58	20.42	14.67	13.66	12.17**
Life activities (5a)	29.50	20.71	10.00	16.33	7.39**
Work activities (5b)^a^	20.75	17.34	7.92	12.41	5.37**
Participation in society (D6)	47.75	13.29	9.92	10.93	21.99**
Zung’s SDS	43.50	12.21	37.36	7.63 **	4.26**

*WHODAS-II* World Health Organization Disability Assessment Schedule-Second Version

^a^Schizophrenia (*n* = 63), normal subjects (*n* = 92)

* *p* < 0.05, ** *p* < 0.001 (*t* tests)

**Table 4:** Pearson’s correlations of disability scores with psychiatric symptoms

	Zung SDS	PANSS positive	PANSS negative	PANSS general psych.	PANSS total
DSS	0.565*	0.402**	0.471**	0.505**	0.617**
D1	0.362**	0.289**	0.275**	0.380**	0.431**
D2	0.397**	0.109	0.294**	0.425**	0.369**
D3	0.286**	0.151	0.228**	0.330**	0.319**
D4	0.342**	0.376**	0.446**	0.269**	0.477**
D5a	0.524**	0.306**	0.313**	0.342**	0.433**
D5b	0.507**	0.184	0.417**	0.192	0.310*
D6	0.465**	0.449**	0.436**	0.485**	0.619**

*Zung SDS* Zung Self-Rating Depression Scale; *PANSS* Positive and Negative Syndrome Scale of schizophrenia; *DSS* disability summary score

* *p* < 0.05, ** *p* < 0.001

The DSS was significantly predicted by depressive symptoms, negative and positive symptoms of schizophrenia, number of active symptoms (relapse) of schizophrenia, and marital status [*F* (5, 94) = 23.90, *p* < 0.001], and this accounted for 56.0 % of the variance (Table 5).

## Discussion

This study investigated disability in patients with schizophrenia using WHODAS-II and compared their level disability with the HC. It also studied the effects of symptoms of schizophrenia (measured with the PANSS) and of depression (measured with Zung’s SDS) on the levels of disability reported by patients with schizophrenia. The results of the study showed that patients with schizophrenia reported significant greater disability when compared with the HC on the total DSS and scores on each of the disability domains of the WHODAS-II except in the “getting around” domain. These findings are consistent with previous observations from the developed countries [15, 16]. The finding on “getting around” domain is understandable given that our sample was taken from stable outpatients. Participation in the society is the disability domain most closely related to symptoms of schizophrenia. The patients also reported the greatest disability severity in this domain reflecting the level of loneliness and psychological distress associated with schizophrenia. It is possible that this may be as a result of several factors such poor social skills, lack of motivation, and self-stigma. Therefore, psychosocial interventions (e.g., social skills training) addressing these factors may help in improving patients community participation as well as their level of disability. In addition, future qualitative studies may want to assess factors that promote and prevent community participation among patients with schizophrenia as this may reveal some factors that standardized instruments did not assess.

**Table 5:** The socio-demographic and clinical variables independently associated with the disability scores by regression analysis

Variables	B	SE	Beta	*t*
Zung SDS	0.412	0.075	0.434	5.462***
PANSS negative	1.396	0.350	0.294	3.993***
Marital status	−5.648	1.867	−0.215	−3.025**
No of active symptoms of schizophrenia (relapse)	1.716	0.549	0.225	3.126**
PANSS positive	0.599	0.240	0.185	2.499*

* p < 0.05, ** p < 0.01, *** p < 0.001

Most of the patients were unmarried and this finding is consistent with that of Lane et al. [31] who reported an over-representation of the unmarried (never married, separated, and divorced) over married in patients with schizophrenia. The higher rate of the unmarried among the patients in this study probably reflects the effects of schizophrenia on interpersonal and intimate relationships [32]. Employment status is an important measure of disability in schizophrenia [33] and the estimates of unemployment in people with schizophrenia in developed countries have been reported to range between 70 and 85 % [34]. In contrast, the unemployment rate in patients with schizophrenia in Ethiopia (a developing country in Africa) was 45.3 % [35] which is close to the 37.0 % reported in this study. The differences in the rates of employment can be explained by the fact that jobs are less complicated in the developing countries when compared to jobs in developed countries [36]. Despite the fact that a good percentage of patients were employed, this study has shown that they were poorly remunerated and this makes them to be economically disadvantaged in the community. Therefore, establishing employment programs for patients may help in reducing their level of disability. In this study, the severity of symptoms of schizophrenia was found to be significantly associated with the patients’ levels of disability. There is evidence that negative symptoms are much more related to disability than positive symptoms in schizophrenia [2, 37, 38]. The severity of positive symptoms correlated with the patients’ abilities to understand and communicate, get along, engage in life activities, and participate in the society, whereas the negative symptoms were significantly related to all the domains of WHODAS-II. Therefore, our study also supported that negative symptoms are much more closely related to disability than positive symptoms of schizophrenia. We found that severity of the depressive symptoms was significantly related to all of the WHODAS-II domains and had the strongest association with the disability summary score. This result is consistent with the findings of other studies [16, 39]. Regression analysis predicting the severity of disability indicated that depressive symptoms, negative and positive symptoms of schizophrenia, number of active symptoms (relapse) of schizophrenia, and marital status were significant predictors. Marital status has been shown to be an independent predictor of outcome in studies both in the developed and developing countries [40, 41]. The finding that the number of episodes of active symptoms (relapses) of schizophrenia is predictor of disability indicates the disruptive effect of psychotic symptoms on the patient’s life. Therefore, prevention of relapses will have significant therapeutic and socio-economic implications. This study has some limitations. First, the study was conducted in a single hospital; therefore, the results obtained may not be representative of the schizophrenic patients’ disability scene in Nigeria. Second, the study was cross-sectional: a longitudinal design would have permitted one to learn more about the nature and course, as well as determine the temporal relationship between symptoms of schizophrenia, depression, and level of disability in patients with schizophrenia. Third, we did not validate the self-reported disability with any objective measure and this may have introduced some response biases. Therefore, combination of self-report and objective measure would have been appropriate and truly capture patients level of disability. However, in rehabilitation, assessment of self-reported disability is a valid method as it can be used to explore and understand complex needs of the patients. In addition, WHODAS-II has been shown to be a valid and reliable self-reported instrument for disability assessment that been used extensively in various countries and across different diseases of which mental illness was included [16]. Fourth, some important socio-demographic variables such as family support and type of residence were not studied.

## Conclusion

The results of this study have shown that our patients with schizophrenia reported greater disability when compared to healthy control in all the domains of WHODAS-II except in the “getting around” domain. They experience the greatest disability in the “participation in the society” domain of WHODAS-II. Therefore, psychosocial interventions such as social skills training targeted towards increasing patients’ involvement in community activities may help in reducing disability in patients with schizophrenia. The level of disability is associated with the severity of depressive symptoms, negative and positive symptoms of schizophrenia, as well as number of relapse and marital status. This study supported that negative symptoms are much more closely related to disability than positive symptoms of schizophrenia. In summary, future multicenter longitudinal studies with large sample size are needed to determine the directionality of the relationship between disability and symptoms of schizophrenia.

## References

[1] Isaac M, Chand P, Murthy P (2007). Schizophrenia outcome measures in the wider international community. Br J Psychiatry.

[2] Alptekin K, Erkoc S, Gogus AK, Kultur S, Mete L, Ucok A, Yazici KM (2005). Disability in schizophrenia: clinical correlates and prediction over 1-year follow-up. Psychiatr Res.

[3] Schneider M, Hurst R, Miller J, Ustun B (2003). The role of environment in the International Classification of Functioning, Disability and Health (ICF). Disabil Rehabil.

[4] World Health Organization (2001). The International Classification of Functioning, Disability and Health (ICF).

[5] World Health Organization (1988). Psychiatric disability assessment schedule (WHODAS).

[6] Srinivasan TN, Rajkumar S, Padmavathi R (2001). Initiating care for untreated schizophrenia initiating care for untreated schizophrenia patients and results of 1 year follow-up. Int J Soc Psychiatry.

[7] Hofer A, Rettenbacher MA, Widschwendter CG, Kemmler G, Hummer M, Fleischhacker WW (2006). Correlates of subjective and functional outcomes in outpatient clinic attendees with schizophrenia and schizoaffective disorder. Eur Arch Psychiatry Clin Neurosci.

[8] McClure MM, Harvey PD (2007). Critical issues in the assessment of disability in schizophrenia. Clin Schizophr Relat Psychos.

[9] Bowie CR, Leung WW, Reichenberg A, McClure MM, Patterson TL, Heaton RK, Harvey PD (2008). Predicting schizophrenia patients’ real world behavior with specific neuropsychological and functional capacity measures. Biol Psychiatry.

[10] Gould F, Sabbag S, Durand D, Patterson TL, Harvey PD (2013). Self-assessment of functional ability in schizophrenia: milestone Achievement and Its Relationship to Accuracy of Self Evaluation. Psychiatry Res.

[11] Bell MD, Bryson G (2001). Work rehabilitation in schizophrenia: does cognitive impairment limit improvement?. Schizophr Bull.

[12] Mueser KT, Salyers MP, Mueser PR (2001). A prospective analysis of work in schizophrenia. Schizophr Bull.

[13] Villalta-Gil V, Vilaplana M, Ochoa S, Haro JM, Dolz M, Usall J, Cervilla J, NEDENA Group (2006). Neurocognitive performance and negative symptoms: are they equal in explaining disability in schizophrenia outpatients?. Schizophr Res.

[14] Rabinowitz J, Levine S, Garibaldi G, Bugarski-Kirola D, Galani C, Kapur S (2012). Negative symptoms have greater impact on functioning than positive symptoms in schizophrenia: analysis of CATIE data. Schizophr Res.

[15] Ertugrul A, Ulug B (2002). The influence of neurocognitive deficits and symptoms on disability in schizophrenia. Acta Psychiatr Scand.

[16] McKibbin CL, Patterson TL, Jeste DV (2004). Assessing disability in older patients with schizophrenia: result from the WHODAS-II. J Nerv Ment Dis.

[17] Gureje O, Ademola A, Olley BO (2008). Depression and disability: comparisons with common physical conditions in the Ibadan study of aging. J Am Geriatr Soc.

[18] Gureje O, Ogunniyi A, Kola L, Afolabi E (2006). Functional disability in elderly Nigerians: results from the Ibadan study of aging. J Am Geriatr Soc.

[19] Uwakwe R, Modebe I (2007). Disability and care-giving in old age in a Nigerian community. Niger J Clin Pract.

[20] Gureje O, Bamidele R (1999). Thirteen-year social outcome among Nigerian outpatients with schizophrenia. Soc Psychiatry Psychiatr Epidemiol.

[21] Adegbaju DA, Olagunju AT, Uwakwe R (2013). A comparative analysis of disability in individuals with bipolar affective disorder and schizophrenia in a sub-saharan African mental health hospital: towards evidence guided rehabilitation. Soc Psychiatry Psychiatr Epidemiol.

[22] World Health Organization (2000). WHO Psychiatric disability assessment schedule (WHO DAS II).

[23] World Health Organisation (1992). International classification of diseases.

[24] Sheehan DV, Lecrubier Y, Harnett-Sheehan K, Amorim P, Janavs J, Weiller E (1998). The Mini International Neuropsychiatric Interview (MINI): the development and validation of a structured diagnostic psychiatric interview. J Clin Psychiatry.

[25] Adewuya AO, Makanjuola ROA (2009). Subjective quality of life of Nigerian schizophrenia patients: sociodemographic and clinical correlates. Acta Psychiatr Scand.

[26] Akinsulore A, Aloba OO, Mapayi BM, Oloniniyi OI, Fatoye FO, Makanjuola ROA (2014). Relationship between depressive symptoms and quality of life in Nigerian patients with schizophrenia. Soc Psychiatry Psychiatr Epidemiol.

[27] Andreasen NC, Pressler M, Nopoulos P, Miller D, Ho B (2010). Antipsychotic Dose equivalents and dose-years: a standardized method for comparing exposure to different drugs. Biol Psychiatry.

[28] Woods SW (2003). Chlorpromazine equivalent doses for the newer atypical antipsychotics. J Clin Psychiatry.

[29] Kay SR, Fyszbern A, Opler LA (1987). The Positive and Negative Syndrome scale (PANSS) for Schizophrenia. Schizophr Bull.

[30] Zung WWK (1965). A self rating depression scale. Arch Gen Psychiatry.

[31] Lane A, Byrne M, Mulvany F, Kinsella A, Waddington JL, Wash D, Larkin C, O’Callaghan E (1995). Reproductive behavior in schizophrenia relative to other mental disorders; evidence for increased fertility in men despite reduced marital rate. Acta Psychiatr Scand.

[32] Zemishlany Z, Weizman A. The impact of mental illness on sexual dysfunction. In: Balon R (ed): sexual dysfunction the brain-body connection. Adv Psychosom Med 2008; 29: pp 89–106. 10.1159/000126626 18391559

[33] Hickling FW, McCallum M, Nooks L, Rodgers-Johnson P (2001). Outcome of first contact schizophrenia in Jamaica. West Indian Med J.

[34] Mulkern VM, Maderscheid RW (1989). Characteristics of community program clients in 1980 and 1984. Hosp Community Psychiatry.

[35] Kebede D, Alem A, Shibre T, Negash A, Fekadu A, Fekadu D, Deyassa N, Jacobsson L, Kullgren G (2003). Onset and clinical course of schizophrenia in Butajira-Ethiopia—a community-based study. Soc Psychiatry Psychiatr Epidemiol.

[36] McGurk S, Meltzer HY (2000). The role of cognition in vocational functioning in schizophrenia. Schizophr Res.

[37] Leisse M, Kallert TW (2000). Social integration and the quality of life of schizophrenic patients in different types of complementary care. Eur Psychiatry.

[38] Patterson TL, Moscona S, McKibbin CL, Davidson K, Jeste DV (2001). Social skills performance assessment among older patients with schizophrenia. Schizophr Res.

[39] Pyne JM, Sullivan G, Kaplan R, Williams DK (2003). Comparing the sensitivity of generic effectiveness measures with symptom improvement in persons with schizophrenia. Med Care.

[40] Jablensky A, Satorius N, Emberg G, Anker M, Kortn A, Cooper JE, Day R, Bertelsen A (1991). Schizophrenia: manifestation, incidence and course in different cultures. A World Health Organization ten-country study. Psychol Med Monogram Suppl.

[41] Padmavathi R, Rajkuma S, Srinivasan TN (1998). Schizophrenic patients who were never treated—a study in an Indian urban community. Psychol Med.

